# Development of a Urine Metabolomics Biomarker-Based Prediction Model for Preeclampsia during Early Pregnancy

**DOI:** 10.3390/metabo13060715

**Published:** 2023-05-31

**Authors:** Yaqi Zhang, Karl G. Sylvester, Bo Jin, Ronald J. Wong, James Schilling, C. James Chou, Zhi Han, Ruben Y. Luo, Lu Tian, Subhashini Ladella, Lihong Mo, Ivana Marić, Yair J. Blumenfeld, Gary L. Darmstadt, Gary M. Shaw, David K. Stevenson, John C. Whitin, Harvey J. Cohen, Doff B. McElhinney, Xuefeng B. Ling

**Affiliations:** 1 College of Automation, Guangdong Polytechnic Normal University, Guangzhou 510665, China; yaqizhang@gpnu.edu.cn; 2 Department of Surgery, Stanford University School of Medicine, Stanford, CA 94305, USA; karls@stanford.edu (K.G.S.); cjchou@stanford.edu (C.J.C.); zhihan01@stanford.edu (Z.H.); 3 mProbe Inc., Palo Alto, CA 94303, USA; bo.jin@mprobe.com (B.J.); james.s@mprobe.com (J.S.); 4 Department of Pediatrics, Stanford University School of Medicine, Stanford, CA 94305, USA; rjwong@stanford.edu (R.J.W.); ivanam@stanford.edu (I.M.); gdarmsta@stanford.edu (G.L.D.); gmshaw@stanford.edu (G.M.S.); dks750@stanford.edu (D.K.S.); cuke@stanford.edu (J.C.W.); punko@stanford.edu (H.J.C.); 5 Department of Pathology, Stanford University School of Medicine, Stanford, CA 94305, USA; rubenluo@stanford.edu; 6 Department of Biomedical Data Science, Stanford University School of Medicine, Stanford, CA 94305, USA; lutian@stanford.edu; 7 Community Medical Centers, UCSF Fresno, Fresno, CA 93722, USA; sladella@communitymedical.org; 8 UC Davis Health, Sacramento, CA 95817, USA; 9 Department of Obstetrics and Gynecology, Stanford University School of Medicine, Stanford, CA 94305, USA; yairb@stanford.edu; 10 Departments of Cardiothoracic Surgery and Pediatrics (Cardiology), Stanford University School of Medicine, Stanford, CA 94305, USA; doff@stanford.edu

**Keywords:** early pregnancy, preeclampsia risk prediction, biomarker, urinary metabolite, LC-MS/MS

## Abstract

Preeclampsia (PE) is a condition that poses a significant risk of maternal mortality and multiple organ failure during pregnancy. Early prediction of PE can enable timely surveillance and interventions, such as low-dose aspirin administration. In this study, conducted at Stanford Health Care, we examined a cohort of 60 pregnant women and collected 478 urine samples between gestational weeks 8 and 20 for comprehensive metabolomic profiling. By employing liquid chromatography mass spectrometry (LCMS/MS), we identified the structures of seven out of 26 metabolomics biomarkers detected. Utilizing the XGBoost algorithm, we developed a predictive model based on these seven metabolomics biomarkers to identify individuals at risk of developing PE. The performance of the model was evaluated using 10-fold cross-validation, yielding an area under the receiver operating characteristic curve of 0.856. Our findings suggest that measuring urinary metabolomics biomarkers offers a noninvasive approach to assess the risk of PE prior to its onset.

## 1. Introduction

Preeclampsia (PE) is a severe hypertensive disorder that can contribute to the mortality and morbidity of pregnant women [1]. PE can cause problems in the liver, kidneys, brain, and blood coagulation system of pregnant women and can also lead to adverse pregnancy outcomes such as poor fetal growth and premature birth [2]. Early treatment with low-dose aspirin can effectively reduce the risk of developing PE [3]. Usually, PE develops after the 20th week of gestation. Therefore, accurate PE prediction before the 20th week could help identify pregnancies at high risk and allow for proactive interventions to improve health outcomes and economic hardships [4].

Considerable progress has been made in using multivariate methods to predict PE [5,6,7]. Relevant factors used for modeling include clinical characteristics [8] and biochemical [9,10] and genetic [11] markers. Extensive research has identified a range of potential biochemical predictors of PE [12,13,14]. Due to the noninvasive collection of urine, urinary analytes could be of great clinical utility for predicting PE risk and subsequent management of pregnancy. Proteomic analysis of urine can also provide valuable information for understanding the pathophysiology of PE [15,16,17,18].

In our recent study, we characterized the baseline weekly profile of the urinary metabolome during a pregnancy uncomplicated by adverse outcomes, which can serve as a high-resolution metabolomic reference for future studies on adverse pregnancy outcomes [19,20]. We hypothesized that deviations from this normal urinary metabolomics profile might identify pregnancies at risk for PE. We used urine samples collected weekly during early pregnancy from pregnant women with PE and non-PE. Using liquid chromatography–mass spectrometry (LC-MS)-based untargeted metabolomics, we identified a panel of metabolic compounds that were highly associated with PE. A predictive model was established to estimate PE risk based on the single urine sample collected in early pregnancy. Our findings suggest that measuring urinary metabolomics biomarkers may provide a noninvasive, cost-effective, and robust approach to assessing PE risk during the first or second trimester of pregnancy. Understanding the functional significance of these PE biomarkers can provide new insights into the pathogenesis and pathophysiology of PE.

## 2. Method

### 2.1. PE Definition

We used the current American College of Obstetrics and Gynecology (ACOG) guideline to define PE [21], which is characterized by hypertension that occurs after 20 weeks of gestation, defined as systolic or diastolic blood pressure of 140 and/or 90 mmHg, respectively, measured on at least two occasions, 4 h to 1 week apart, and proteinuria, which can be indicated by 300 mg of protein in a 24 h urine collection, a protein/creatinine ratio of at least 0.3 (each measured as mg/dL), or, if these measurements are not readily available, a random urine specimen containing 1+ protein by dipstick. In the absence of proteinuria, a diagnosis of preeclampsia can still be made if there is evidence of thrombocytopenia (platelet count less than 100,000/mL), impaired liver function (elevated blood levels of liver transaminases to twice the normal concentration), new development of renal insufficiency (elevated serum creatinine greater than 1.1 mg/dL), pulmonary edema, or new-onset cerebral or visual disturbances. Early- and late-onset PE are distinguished based on whether the diagnosis is made before or after 34 weeks of gestation, respectively.

The severe PE was also diagnosed based on criteria of ACOG, including systolic blood pressure of 160 mmHg or more, or diastolic blood pressure of 110 mmHg or more on two occasions at least 4 h apart (unless antihypertensive therapy is initiated before this time), thrombocytopenia (platelet count less than 100,000 × 10^9^/L), impaired liver function that is not accounted for by alternative diagnoses and as indicated by abnormally elevated blood concentrations of liver enzymes (to more than twice the upper limit for normal concentrations), or by severe persistent right upper quadrant or epigastric pain unresponsive to medications. We classified all PE except for severe PE as mild PE.

### 2.2. Cohort Construction

To develop and validate the PE model, we recruited 60 pregnant women who gave birth at Stanford Health Care (SHC) (Stanford, CA, USA). Of these, 19 had full-term pregnancies without PE, 13 had full-term pregnancies with PE, 21 had preterm pregnancies without PE, and 7 had preterm pregnancies with PE. All patients had singleton pregnancies. The research was conducted without patient involvement, and patients were not consulted on the study design or invited to comment on the results. Ethics committees at Stanford University approved the study (IRB # 21956), and all participants provided written informed consent. Patients were not involved in the writing or editing of this document for readability or accuracy.

### 2.3. Samples

**Figure 1:**
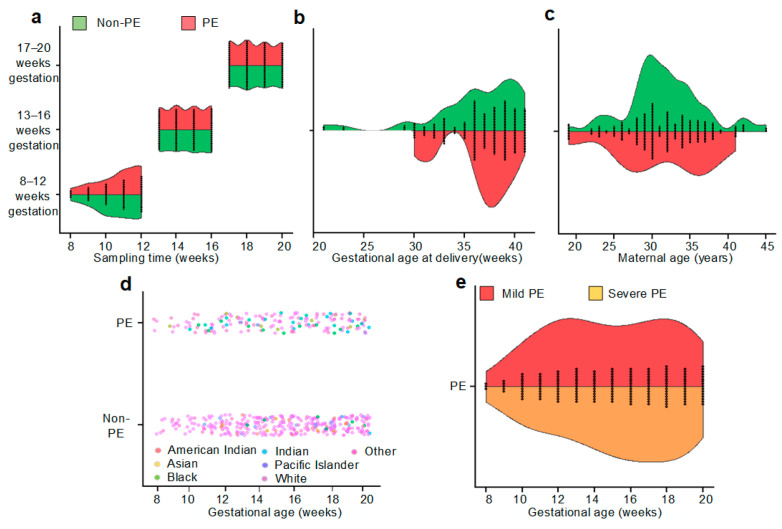
Comparisons of sample, maternal, and pregnancy characteristics for cases of PE and controls (non-cases) in the study cohort. In this study, we treated each longitudinal sampling as an independent analysis unit while considering the temporal gestational age association. Panel (**a**) shows the sample collection time across gestation, while panel (**b**) presents the gestational age at delivery of samples. Panels (**c**,**d**) illustrate the maternal characteristics of age, and race of the PE and non-PE urine sampling groups. Panel (**e**) shows the PE patients’ severity distribution in the PE urine samples.

The urine was retrospectively collected and aliquoted and stored in −80 °C immediately. No freeze thaw cycle was allowed and all urine samples for the analysis were prepared fresh directly from −80 °C aliquots. Urine samples (*n* = 478) were longitudinally collected from 60 pregnant women of various races, geographic locations, and socioeconomic statuses. The distributions of sample, maternal, and pregnancy characteristics for cases (PE) and controls (non-PE) are shown in Figure 1. The time range of sample collection was between the 8th and 20th week of gestation. A total of 163 urine samples from 19 full-term pregnancies without PE were collected in the first (*n* = 49) and second (*n* = 114) trimesters, and 109 urine samples from 13 full-term pregnancies with PE were collected in the first (*n* = 35) and second (*n* = 74) trimesters. A total of 153 urine samples from 21 preterm pregnancies without PE were collected in the first (*n* = 49) and second (*n* = 104) trimesters. A total of 53 urine samples from seven preterm pregnancies with PE were collected in the first (*n* = 22) and second (*n* = 31) trimesters (Figure S1). Pregnant women were of different ages, races, and PE severities (Table 1).

### 2.4. Urinary Metabolite Extraction and Global Liquid Chromatography Mass Spectrometry (LC-MS/MS) Analysis

The urinary metabolite extraction and LC-MS/MS global metabolomics profiling were performed using the precipitation-based approach, as previously described [22]. Briefly, 10 µL of urine was mixed with 100 µL of methanol containing 5 μg/mL of 13C5, 15N-l-proline, 13C6-l-arginine, and D5-l-glutamine. The extract was vortexed and centrifuged, and 90 µL of the supernatant was collected for global metabolomics analysis.

QC urine samples were analyzed repetitively to assess data quality. Metabolomics LCMS with quality control (QC) samples at regular intervals, such as every 10 samples in our study, is crucial for ensuring the reliability and accuracy of the metabolomics profiling data. To generate a quality control (QC) sample, 10 μL of each of the PE and non-PE urine samples were pooled into a single tube [23], representative of the entire study cohort. These samples are analyzed alongside the study samples to monitor the performance and stability of the LCMS instrument and the overall analytical process. Including urine QC samples in metabolomics LCMS experiments serves several important purposes. First, QC samples provide a reference standard that allows for the evaluation of the analytical variability and reproducibility. By comparing the measurements of metabolites in QC samples across different runs or batches, researchers can identify any technical variations and assess the overall quality of the data. Secondly, the analysis of QC samples helps in monitoring the stability and performance of the LCMS instrument over time. Any drift or instrumental issues can be detected by examining the consistency of the measurements in QC samples. If deviations are observed, corrective actions can be taken to address the problem and ensure data integrity. Thirdly, QC samples are also used for data normalization and correction of batch effects. Metabolomics profiling studies often involve the analysis of multiple batches or runs, which can introduce unwanted technical variation. By comparing the measurements in QC samples across different batches, it is possible to adjust and normalize the data, reducing batch effects and improving the accuracy of the statistical analyses. Overall, the inclusion of urine QC samples in our longitudinal metabolomics LCMS experiments allows for the assessment of data quality, instrument performance monitoring, and data normalization. It enhances the reliability and reproducibility of the metabolomics profiling data, enabling more robust and meaningful interpretation of the results.

All samples were analyzed using an LC metabolomics platform that employed hydrophilic interaction chromatography (HILIC) with a column size of 2.1 mm × 100 mm × 3.5 μm. The global MS analysis was performed using a Vanquish UHPLC system coupled to Q Exactive plus and Q Exactive HF hybrid Quadrupole-Orbitrap mass spectrometers manufactured by ThermoFisher in San Jose, CA, USA. This LC-MS platform allowed for comprehensive profiling of the metabolites present in the samples, enabling the identification and quantification of a wide range of compounds.

### 2.5. Data Preprocessing and Statistics

The raw data obtained from the MS analysis were subjected to preprocessing to create a data matrix that represented the relative abundance of metabolites across all the samples. To ensure data accuracy and minimize variation, a robust QC-based locally estimated scatterplot smoothing (LOESS) signal correction method was applied. This involved fitting a LOESS curve to the signal responses obtained from QC replicates, allowing for independent correction of each feature. The R xcms package was utilized for this preprocessing step. Furthermore, to mitigate batch effects, the metabolite values in each sample were normalized using the median values obtained from the QC samples. This normalization process helped to enhance the comparability and reliability of the data across different batches or runs.

Urine molecular profiling presents a challenge due to the influence of biological dilution, which can be influenced by factors like diet, hydration status, and kidney function, leading to significant variations in urine analyte concentrations. To address this challenge and ensure accurate analysis, we implemented two normalization strategies to account for differences in urine concentration among samples. Firstly, we utilized the probabilistic quotient method, as described in reference [24], to adjust for the variations in urine concentration. This method provides a statistical approach to normalize the data and account for the dilution effect, enabling more reliable comparisons across samples. Additionally, creatinine was employed as a normalization factor in this study, following our previous approach outlined in reference [22]. Creatinine was chosen due to its stability and consistent excretion rate in urine, making it a suitable marker for normalization purposes. By using creatinine as a normalization factor, we aimed to further mitigate the impact of biological dilution and enhance the accuracy of our urine molecular profiling analysis. These normalization strategies were crucial in overcoming the challenges associated with biological dilution, allowing us to obtain more reliable and meaningful insights from the urine molecular profiling data.

For downstream analyses, we implemented specific criteria to ensure the quality and reliability of the metabolomics features. Features that exhibited a coefficient of variation (CV) of less than or equal to 20% in the QC samples, as well as those with missing values in less than or equal to 30% of the samples, were selected for further analysis. To identify metabolites that showed differential expression between groups, we employed the DESeq2 package. This approach allowed us to statistically assess the significance of differential expression and identify metabolites that were associated with the condition of interest. Furthermore, to characterize the metabolomics profiling of samples collected during the first and second trimesters, we employed partial least squares discriminant analysis (PLS-DA). This multivariate analysis technique enabled us to explore the patterns and differences in metabolite profiles between the two trimesters. To select metabolites that were significantly associated with preeclampsia (PE), we applied multiple criteria. These included false discovery rates (FDRs), fold changes, and correlation analysis using Spearman’s rank correlation. By considering these criteria, we identified metabolites that exhibited significant associations with PE. Additionally, we assessed the significance of metabolic pathways using the one-sided Fisher exact test and KEGG pathways [25]. This allowed us to gain insights into the biological processes and pathways that were perturbed in relation to PE.

All statistical analyses were conducted using various R packages [26], providing a robust and comprehensive framework for analyzing and interpreting the metabolomics data in this study.

### 2.6. Metabolite Identification and Metabolite-Based Modeling

Metabolite identification in this study followed a rigorous approach based on tier one or two identification guidelines outlined by the Metabolomics Standards Initiative (MSI) [27]. We utilized chemical standards to aid in the identification process. Tandem MS/MS data obtained from urine samples using the Thermo Q Exactive plus instrument were utilized to generate MS1/MS2 profiles. These profiles were then subjected to database searching using publicly available resources such as HMDB, MoNA, MassBank, METLIN, and NIST. To confirm the identity of potential biomarker candidates, we obtained reference compounds of the metabolites of interest. These reference compounds were then subjected to a tier one identification approach, which involved comparing their retention times and MS1/MS2 patterns with those obtained using the same LC-MS/MS protocol as used in the study. By employing this comprehensive identification strategy, we aimed to ensure the accuracy and reliability of the metabolite identifications in our study, strengthening the validity of the biomarker candidates we identified.

In order to predict the risk of developing PE, we developed an XGBoost model utilizing the metabolic features identified in our study. The model’s parameters were carefully selected through a rigorous process of 10-fold cross-validation using our dataset. To evaluate the performance of the model, we employed receiver operating characteristic curves (ROCs) and calculated the corresponding areas under the curve (AUCs). These metrics provided valuable insights into the prediction accuracy of our model. To further analyze the risk stratification capabilities of the model, we compared the probabilities of PE between low-risk and high-risk categories using Kaplan–Meier survival analyses. This allowed us to assess the differences in PE occurrence between these two groups. Additionally, we examined the trends of changes in the significant indicators throughout pregnancy, their correlations with maternal health, and their potential associations with other diseases. These discussions provided a comprehensive understanding of the dynamics of these indicators and their relevance in the context of PE. By incorporating these analytical approaches, we aimed to not only develop a robust prediction model for PE risk but also gain insights into the underlying mechanisms and potential clinical implications of the identified indicators.

## 3. Results

### 3.1. A Unique PE-Associated Metabolomics Pattern and Metabolic Pathway Analyses

**Figure 2:**
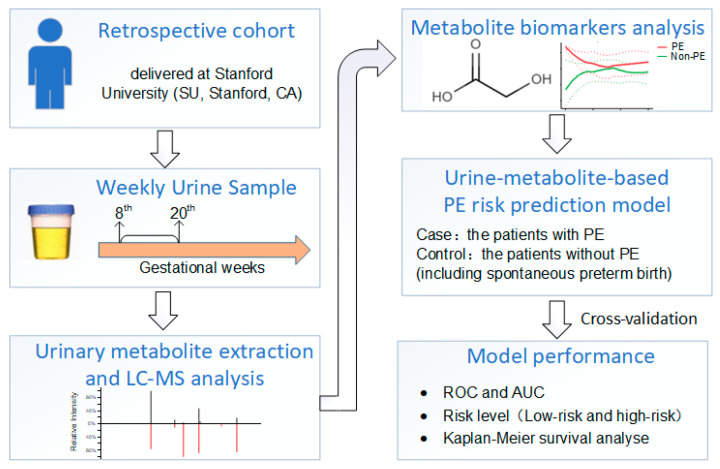
Schematic diagram of the overall study workflow.

**Figure 3:**
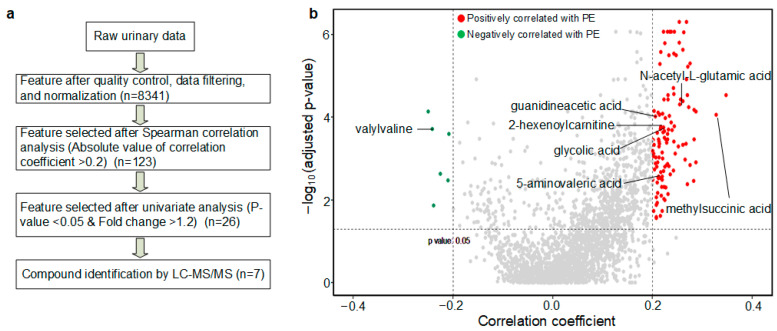
The feature-selection process is depicted in (**a**), and the features selected via Spearman correlation and *p*-value are shown in (**b**). The *y*-axis displays the negative logarithm (base 10) of the *p*-value adjusted by FDR, with features altered during pregnancy gestational age, while the *x*-axis displays the Spearman correlation coefficients between the features and PE. Red dots represent features that are positively correlated with PE, while green dots represent features that are negatively correlated with PE. The names of the seven significant metabolites, that were later structurally determined, are listed.

The study workflow is illustrated in Figure 2; it involved several key steps. Initially, LC-MS metabolomics profiling was performed, resulting in the identification of 8341 metabolic features. Subsequently, quality control (QC), data filtering, and normalization procedures were applied (Figure 3a) to ensure the reliability and consistency of the data.

To gain a comprehensive understanding of the global metabolic patterns associated with PE and non-PE pregnancies during the 8th to 20th week of gestation, an unsupervised clustering algorithm known as partial least squares discriminant analysis (PLS-DA) was employed (Figure S2). This analysis revealed distinct metabolomics patterns that differentiated between the two pregnancy groups.

**Figure 4:**
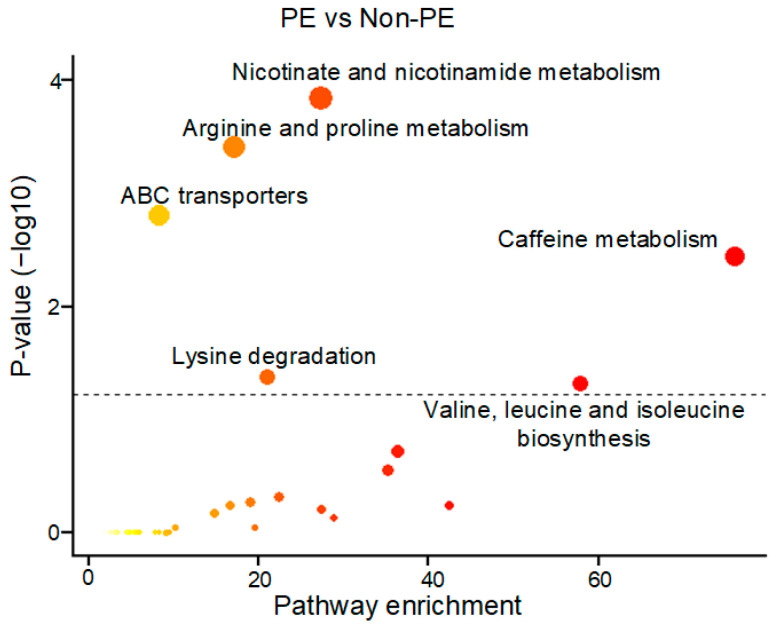
The KEGG metabolic pathway enrichment analyses were performed to compare the maternal urine metabolomes of the PE and non-PE cohorts. The resulting plot displays the −log10(*p*-value) of metabolic pathways on the vertical axis (*y*-axis), while the corresponding pathway enrichment is shown on the horizontal axis (*x*-axis). Pathways that achieved a *p*-value of <0.05 are highlighted as red circles.

Furthermore, pathway enrichment analyses were conducted to identify significant pathways (*p* < 0.05) associated with PE pregnancies (Figure 4). Notably, several pathways emerged as being significantly linked to PE, including nicotinate and nicotinamide metabolism, arginine and proline metabolism, ABC transporters, caffeine metabolism, lysine degradation, and valine and leucine biosynthesis. These findings shed light on the potential metabolic dysregulations underlying the development of PE.

By employing these analytical approaches and pathway enrichment analyses, we were able to unravel unique metabolomic patterns and identify key pathways associated with PE pregnancies during the specified gestational period. These findings contribute to our understanding of the metabolic alterations associated with PE and provide valuable insights for future research and clinical interventions.

### 3.2. PE Predictive Metabolomics Biomarkers

A comprehensive analysis of the metabolomics data led to the identification of 26 maternal metabolite features that showed significant associations with PE. These associations were determined through univariate analysis of metabolic features, comparing PE with non-PE urinary metabolomes (Figure 3b). To be considered significant, these metabolites had to meet the criteria of having a *p*-value less than 0.05, a case/control median fold change greater than 1.2, and an absolute value of Spearman correlation coefficient greater than 0.2.

**Figure 5:**
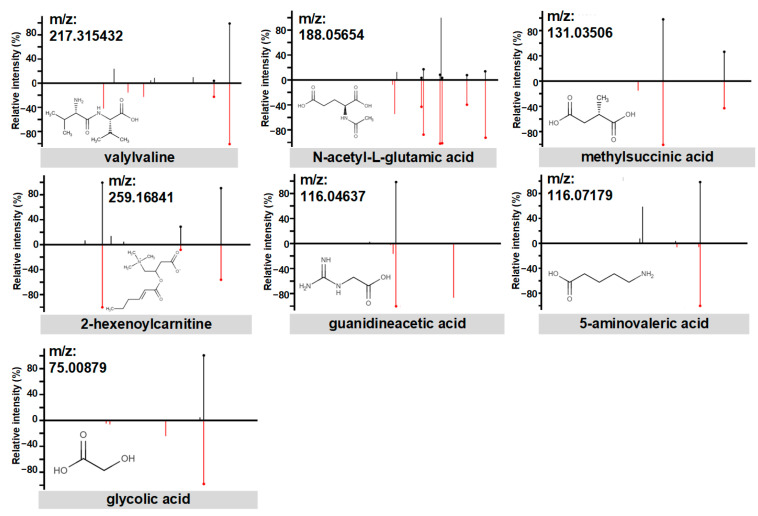
The structural identification of the seven metabolites by MS/MS fragmentation against reference compound standards. Measured MS/MS spectral fragmentation profiles (top, black lines) matching chemical standards (bottom, red lines).

Among the identified metabolites, seven compounds were subjected to further analysis through LC-MS/MS profiling and reference compound matching analyses. These seven compounds, namely guanidineacetic acid, 2-hexenoylcarnitine, glycolic acid, valylvaline, methylsuccinic acid, N-acetyl-L-glutamic acid, and 5-aminovaleric acid, exhibited positive model importance in the XGBoost multivariate modeling analysis. The identity of these metabolites was confirmed through MS/MS analyses, comparing them with reference compound standards (Figure 5).

**Figure 6:**
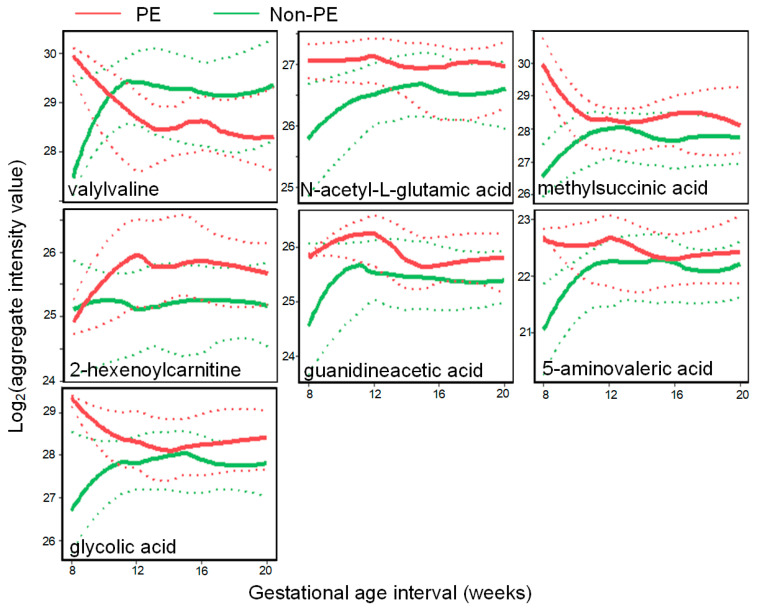
PE metabolomics biomarkers with unique urinary abundance patterns along gestation. The mean levels (solid lines) with the 95% confidence intervals (CIs, dotted lines) of the metabolite changes as a function of the gestational age are shown for PE (red) and non-PE (green) births.

Moreover, when comparing PE and non-PE urine samples, distinct gestational age patterns were observed for these seven metabolites (Figure 6). These patterns provided further insights into the dynamic changes in metabolite levels throughout pregnancy and their potential relevance to the development of PE.

The combined findings from the univariate analysis, structural identification, multivariate modeling, and gestational age patterns highlighted the significance of these seven metabolites in relation to PE. These findings enhance our understanding of the metabolic alterations associated with PE and provide a foundation for the development of a metabolomics panel for early outcome prediction.

### 3.3. Performance of the PE Prediction Model

A predictive model for PE was developed using the XGBoost algorithm based on seven maternal urine metabolites. The accuracy of the model in predicting PE during the 8th to 20th week of gestation was assessed. The importance of each compound in the model is depicted in Figure S3.

**Figure 7:**
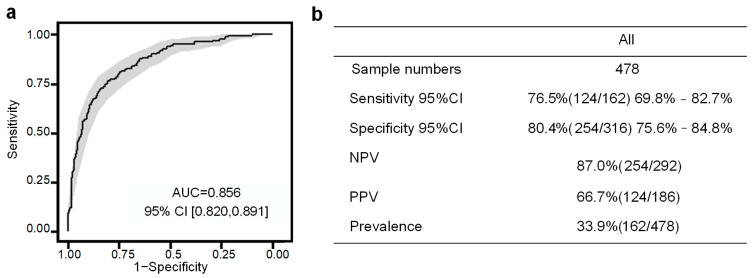
Evaluation of PE prediction with SHC cohort. (**a**) AUCs and (**b**) confusion matrix performance of the PE prediction model.

**Figure 8:**
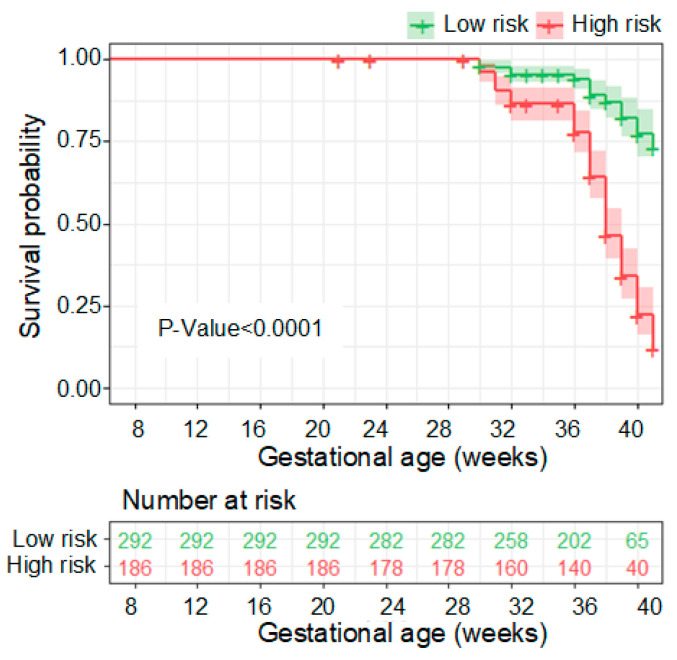
Kaplan–Meier analyses of deliveries of low- and high-risk PE pregnancies. The vertical axis (*y*-axis) displays the population proportion in the two risk subgroups who were not diagnosed with PE over time, and the horizontal axis (*x*-axis) displays the corresponding gestational age.

In Figure 7a, the performance of the seven-compound panel in predicting PE is shown. The model achieved an area under the curve (AUC) of 0.856, indicating good predictive capability, as determined through 10-fold cross-validation in the SHC cohort. Furthermore, at a sensitivity of 76.5%, the PE risk predictive model demonstrated a positive predictive value (PPV) of 66.7% (Figure 7b). These results highlight the potential of the model to accurately identify individuals at risk of developing PE during early pregnancy (Figure 8).

The utilization of the XGBoost algorithm and the inclusion of the seven maternal urine metabolites contribute to the predictive power of the model. These findings have implications for early risk assessment and intervention strategies, ultimately aiming to prevent or reduce the impact of PE on maternal and fetal health.

Instead of employing the probabilistic quotient normalization method [24], we adopted an internal urine creatinine-based approach [22] to normalize the seven maternal urine metabolite-based biomarkers in the development of our PE predictive model. Figure S4 illustrates the distribution of the data for the seven metabolites after applying different normalization strategies. The model importance of each compound is presented in Figure S5.

**Figure 9:**
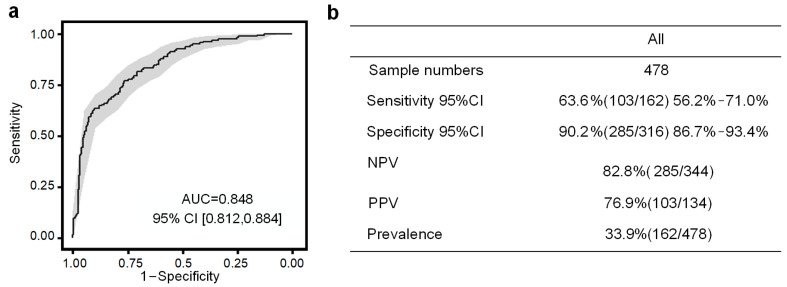
Evaluation of PE prediction with SHC cohort after the creatine normalization. (**a**) AUCs and (**b**) confusion matrix performance of the PE prediction model.

**Figure 10:**
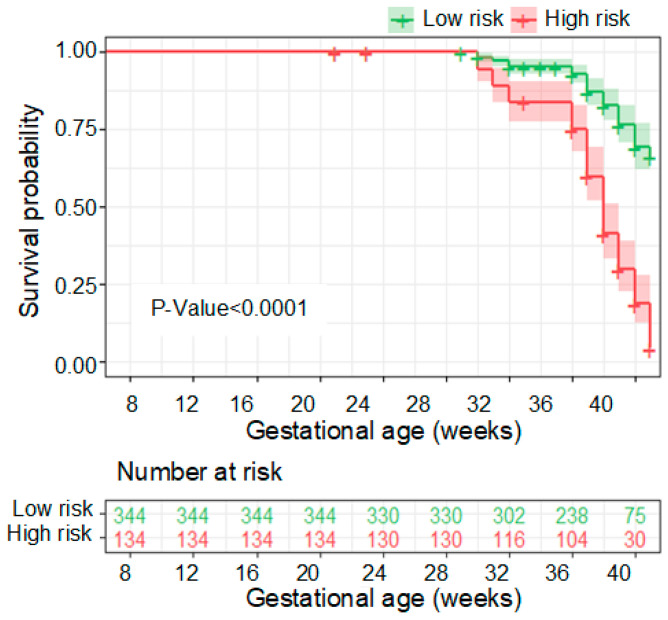
Kaplan–Meier analyses of deliveries of low- and high-risk PE pregnancies after the creatine normalization. The vertical axis (*y*-axis) displays the population proportion in the two risk subgroups who were not diagnosed with PE over time, and the horizontal axis (*x*-axis) displays the corresponding gestational age.

In Figure 9a, the model performance remains consistent, with an area under the curve (AUC) of 0.848, as determined by 10-fold cross-validation in the SHC cohort. At a sensitivity of 63.6% and a specificity of 90.2%, our PE risk predictive model achieves a positive predictive value (PPV) of 76.9% (Figure 9b). Furthermore, the survival curve generated using this model demonstrates significant differences in the PE diagnosis curve between the two risk populations. This further confirms the effectiveness of the model established using these seven biomarkers in assessing the risk stratification of future PE events (Figure 10). These findings highlight the potential clinical utility of the model in predicting and managing PE, enabling timely interventions to improve maternal and fetal outcomes.

## 4. Discussion

### 4.1. Summary of Main Findings

The primary objective of this paper is to estimate the risk of PE during early pregnancy and devise preventive measures to reduce its symptoms through early intervention. In line with current recommendations, low-dose aspirin (81 mg/day) prophylaxis is advised for women at high risk of PE, commencing between 12 and 28 weeks of gestation (preferably before 16 weeks) and continued until delivery. To address this objective, we specifically selected the time frame of the 8th to 20th week of gestation for sampling. By evaluating the performance of our predictive model, we can assess the efficacy of biomarkers obtained during this gestational period in predicting the occurrence of PE, as detailed in this article.

We conducted a study at SHC involving a cohort of 60 pregnant women to investigate whether the urine metabolome during pregnancy could serve as a predictive tool for the onset of PE. Through urine metabolomics profiling, we identified a total of 8341 metabolic features. Among them, we identified 26 metabolites that showed significant association with the risk of PE. Further analysis enabled us to determine the structures of seven of these metabolites. Leveraging these seven metabolites, we developed an early risk prediction model for PE using urine samples collected between the 8th and 20th weeks of pregnancy.

To validate the robustness of our model, we employed a 10-fold cross-validation approach, which provided support for the notion that longitudinal analysis of urine metabolites in early pregnancy can effectively predict the onset of PE. This approach offers a noninvasive and cost-effective means of assessing PE risk. We also explored two urine normalization methods, namely the probabilistic quotient [24] and the internal urine creatinine [22]-based approach, which yielded comparable modeling performances in predicting the occurrence of PE.

### 4.2. Biological Implications of PE Biomarkers

The LCMS profiling of urine metabolite biomarkers conducted in this study has provided insights into the expression patterns of these biomarkers in pregnancies with PE and those without (non-PE). Interestingly, we observed considerable overlap in the urine expression patterns between the two groups. This finding indicates that relying solely on a single time-point measurement of metabolite biomarkers or a single metabolic biomarker may not be sufficient for early and accurate prediction of PE.

The observed overlap in metabolite biomarker expression aligns with the current understanding that PE is a complex and multifactorial disease. It involves a range of underlying factors that contribute to its development. Therefore, capturing the dynamic nature of PE requires considering multiple time points and a comprehensive panel of metabolite biomarkers.

Our findings shed light on the intricate processes involved in the pathogenesis of PE. These processes extend beyond specific time points or gestational ages, which explains the observed overlap in metabolite biomarker expression between PE and non-PE pregnancies. To achieve reliable and precise prediction of PE, it is crucial to consider the complexity and multifactorial nature of the disease, as well as the dynamic changes in metabolite biomarkers over the course of pregnancy.

Incorporating longitudinal sampling and analysis of metabolite biomarkers throughout pregnancy holds great promise in providing valuable insights into the evolving risk of developing PE. This approach enables us to monitor the dynamic changes in metabolite biomarkers over time, allowing for timely interventions and improved maternal and fetal outcomes.

Among the seven PE biomarkers identified in our study, six showed a positive association with PE outcomes, while one exhibited a negative association. This suggests that these metabolite biomarkers may play a role in the pathogenesis of PE and could serve as potential indicators of disease progression.

Intriguingly, our analysis of the metabolite–metabolite interaction network, as shown in Figure S6, revealed potential functional relationships among guanidineacetic acid, N-acetyl-L-glutamic acid, and 5-aminovaleric acid. These metabolites may interact with each other in complex physiological processes relevant to PE.

Previous studies have explored the relationship between 5-aminopentanoic acid and PE. This metabolite, which is a lysine degradation product, has been implicated in various physiological processes, including vascular function and blood pressure regulation, which are relevant to the pathophysiology of PE. Elevated levels of 5-aminopentanoic acid in biofluids may indicate conditions such as bacterial overgrowth or endogenous tissue necrosis [28].

Furthermore, excessive amounts of N-acetyl amino acids have been observed in the urine of individuals with aminoacylase I deficiency, a genetic disorder associated with kidney damage, cardiovascular disease, and neurological deficits [29]. Guanidoacetic acid, on the other hand, serves as a biomarker for an inborn metabolic disorder called guanidinoacetate methyltransferase (GAMT) deficiency [30]. Additionally, certain medium-chain acylcarnitines, including 2-hexenoylcarnitine, can serve as useful markers for inherited disorders of fatty acid metabolism [31]. Increased urinary levels of methylsuccinic acid, along with ethylmalonic acid, are the main measurable biochemical features in ethylmalonic encephalopathy, an autosomal recessive disorder [32].

By uncovering these associations and providing insights into the underlying metabolic processes, our study contributes to a better understanding of the potential mechanisms involved in the development of PE.

### 4.3. Comparison with Prior Work and Limitations

The current study builds upon our previous work [22], which demonstrated that the weekly urinary metabolomics profile during pregnancy serves as a high-resolution molecular reference for future studies of adverse pregnancy outcomes. Our PE risk prediction model differs from previous studies in terms of predictors, sampling matrices, and test methods [6,7,8,12,13,15,33,34,35]. We explored the use of either the probabilistic quotient [24] or the internal urine creatinine [22]-based approach to normalize our urine biomarkers to overcome the challenge of biological dilution in urine molecular profiling. Given that there is a limited number of studies using urine samples collected weekly starting from the first trimester to predict PE risk, our approach is innovative and has technological advantages.

However, it is important to acknowledge the limitations of our study. Firstly, it was retrospective in nature, and the study population was limited to a single geographic region (CA, USA) without a verification group. This restricts the generalizability of our findings and calls for caution when applying them to other populations or regions. In addition, the feature-selection process did not incorporate cross-validation in conjunction with XGBoost learning, which could have led to an overestimation of our model’s performance. Secondly, the diversity of race and age in our case and control subgroups was relatively narrow, which may not fully represent the demographics of the general population or other regions. It is crucial to include a broader range of racial and ethnic backgrounds to ensure the robustness and applicability of our findings across diverse populations. Thirdly, we treated each longitudinal sampling as an independent analysis unit while considering the temporal association. This approach may have resulted in the loss of within-subject correlation, suboptimal utilization of high-resolution urine sampling data, inaccurate estimation of gestational effects, and flawed modeling of temporal trends in PE outcomes. To address these issues, more appropriate statistical methods, such as mixed-effects models, generalized estimating equations (GEEs), or time-series analysis, should be employed to explicitly account for within-subject correlation and temporal dependencies. This would provide more accurate parameter estimation, improved inference, and better modeling of temporal trends. Fourthly, while our model demonstrated the ability to predict PE risk with a single urine sample during early pregnancy, the timing of onset and the underlying biology of PE require further extensive investigation. It is important to conduct larger prospective multi-center cohort studies that encompass a diverse racial and ethnic population to validate the clinical utility of our urinary metabolite panel in predicting PE.

Although our study has provided valuable insights into the potential of urine metabolite biomarkers for predicting PE, it is crucial to address these limitations through further research. By incorporating more diverse populations, utilizing robust statistical methods, and conducting prospective studies, we can enhance the validity and clinical applicability of our findings in the prediction and understanding of PE.

### 4.4. Advantage of Urine Testing of PE Risk Prediction

The study highlights the clinical importance of utilizing urinary metabolomics profiling in pregnant women as a means of predicting PE. By identifying a panel of urine metabolites during early pregnancy, we have established a noninvasive test that can effectively predict the risk of developing PE. This approach offers an alternative option for monitoring the fetal growth environment, particularly for women who have limited access to traditional clinical utilities such as ultrasound analysis.

Nevertheless, further investigations are necessary to explore the correlation between the risk score obtained from our model and the timing of onset for PE. By understanding the relationship between the risk score and the actual development of PE, we can refine our predictive capabilities and enhance the precision of early intervention strategies. Notably, the advantage of noninvasive sampling with high resolution enables the early identification of high-risk patients for PE. This timely identification empowers healthcare providers to initiate interventions, such as low-dose aspirin, in order to mitigate potential complications associated with PE.

In summary, our study points to the clinical significance of urinary metabolomics profiling in pregnant women as a valuable tool for predicting PE. This approach offers a noninvasive alternative, particularly beneficial for individuals with limited access to traditional monitoring methods. However, further research is required to investigate the relationship between the risk score and the timing of onset, ultimately refining our predictive capabilities. The ability to identify high-risk patients early on allows for timely interventions, such as low-dose aspirin, which can help mitigate potential complications associated with PE.

## 5. Conclusions

Our study demonstrates the efficacy of maternal urinary metabolomics profiling as a noninvasive approach for predicting the risk of PE during the first and second trimesters of pregnancy. The robustness of our modeling, which incorporates diverse racial groups and maternal ages, coupled with the simplicity of sample acquisition, enhances its potential for clinical development and practical application.

This prediction tool serves as a valuable reference for early diagnosis of PE in pregnant women, enabling timely and targeted interventions to ensure the safety of both the mother and the fetus. By monitoring the biomarkers and exploring potential enrichment pathways of metabolites, we gain new insights into the pathophysiological mechanisms underlying abnormal fetal development and adverse pregnancy conditions, including PE.

The use of maternal urinary metabolomics profiling offers a promising avenue for further research and clinical implementation. Its noninvasive nature and ability to provide valuable predictive information make it a valuable tool for healthcare providers in managing and monitoring high-risk pregnancies. Through ongoing studies and investigations, we can continue to advance our understanding of PE and develop effective strategies to improve pregnancy outcomes.

## Figures and Tables

**Table 1:** Demographics of the subjects enrolled at SHC.

	PE (*n* = 20)	Non-PE (*n* = 40)	*p*-Value
Number of samples	162	316	
Cohort, *n* (%)			<0.001
Full-term with PE	13 (65)	0 (0)	
Pre-term with PE	7 (35)	0 (0)	
Pre-term without PE	0 (0)	21 (52.5)	
Full-term without PE	0 (0)	19 (47.5)	
Age, mean (IQR)	35.1 (27.8, 36.2)	32.1 (29, 35)	0.84
GA at delivery, mean (IQR)	37 (36, 39)	36 (33.8, 39)	0.65
Hypertensive disorder, *n* (%)			<0.001
Mild PE	10 (50)	0 (0)	
Severe PE	10 (50)	0 (0)	
Non-PE	0 (0)	40 (100)	
Race, *n* (%)			0.21
American Indian	0 (0)	2 (5)	
Asian	2 (10)	1 (2.5)	
Black	1 (5)	1 (2.5)	
Indian	2 (10)	1 (2.5)	
Pacific Islander	0 (0)	1 (2.5)	
White	9 (45)	28 (70)	
Other	6 (30)	6 (15)	
Total number of previous pregnancies, mean (SD)	1.1 (2.0)	1.9 (2.2)	0.19

Values are mean ± SD or numbers (percentages). SD: Standard deviation; GA: Gestational age; IQR: Interquartile Range.

## Data Availability

The datasets used or analyzed during the current study are available from the corresponding author on reasonable request due to privacy.

## Supplementary Materials

The following supporting information can be downloaded at: https://www.mdpi.com/article/10.3390/metabo13060715/s1, Figure S1: The sample distributions in the cohort. Charts of urine collection timepoints for the SHC cohort. Each line represents an individual patient. Diamonds and triangles indicate sample collection and delivery dates, respectively. Figure S2: Distribution of individual samples in partial least-squares discriminant analysis based on 8341 features as a function of the PE outcomes. The two orthogonal components with most of the inertia are shown. Figure S3: The importance of the seven metabolites in our PE risk model with the probabilistic quotient normalization method. Figure S4: The distribution of the seven metabolites data after different normalization strategies. (a) shows the intensity value of metabolites in PE and non-PE populations used the probabilistic quotient method; (b) shows the intensity value of metabolites in PE and non-PE populations after creatinine correction. Figure S5: The importance of the seven metabolites in our PE risk model with the creatinine normalization method Figure S6: The metabolite-metabolite interaction network with the chemical-chemical associations.
