# Supplementary figures and images for: Development of a Urine Metabolomics Biomarker-Based Prediction Model for Preeclampsia during Early Pregnancy
Source: Metabolites. 2023 May 31;13(6):715. doi: 10.3390/metabo13060715 (PMC10301596; doi:10.3390/metabo13060715)

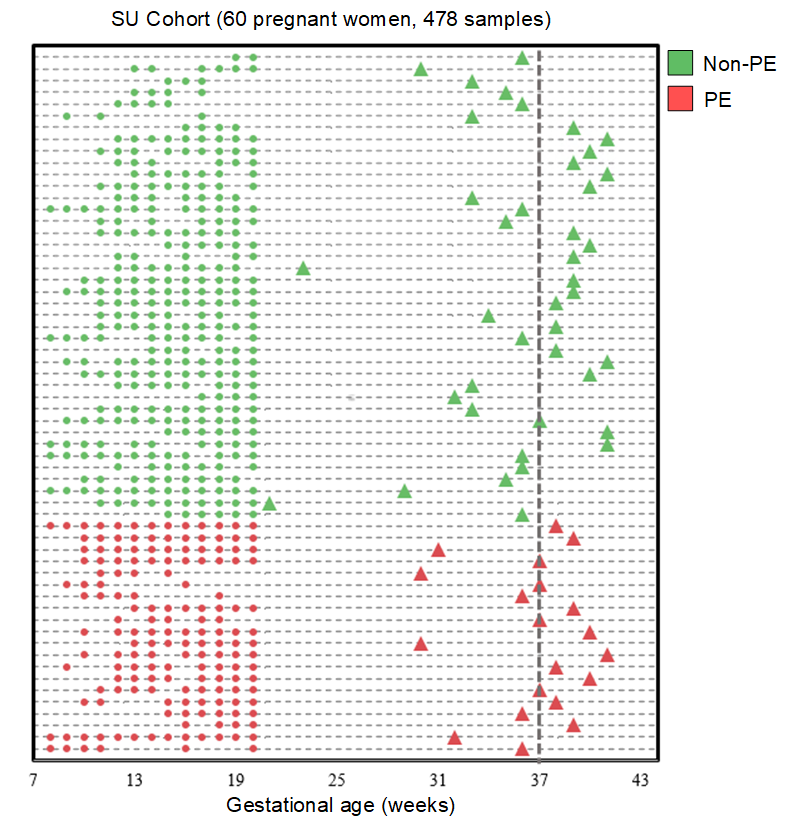

Supplement: Supplementary file 1 [file metabolites-13-00715-s001.zip › Supplementary File/Figure S1.png]

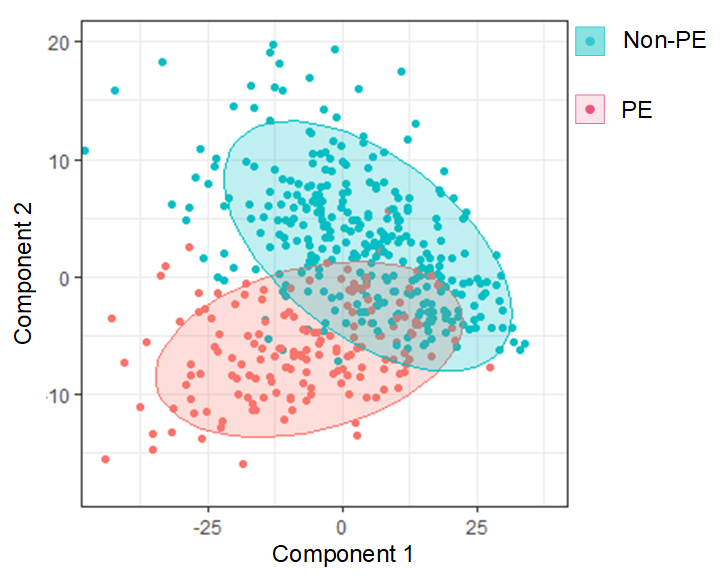

Supplement: Supplementary file 1 [file metabolites-13-00715-s001.zip › Supplementary File/Figure S2.png]

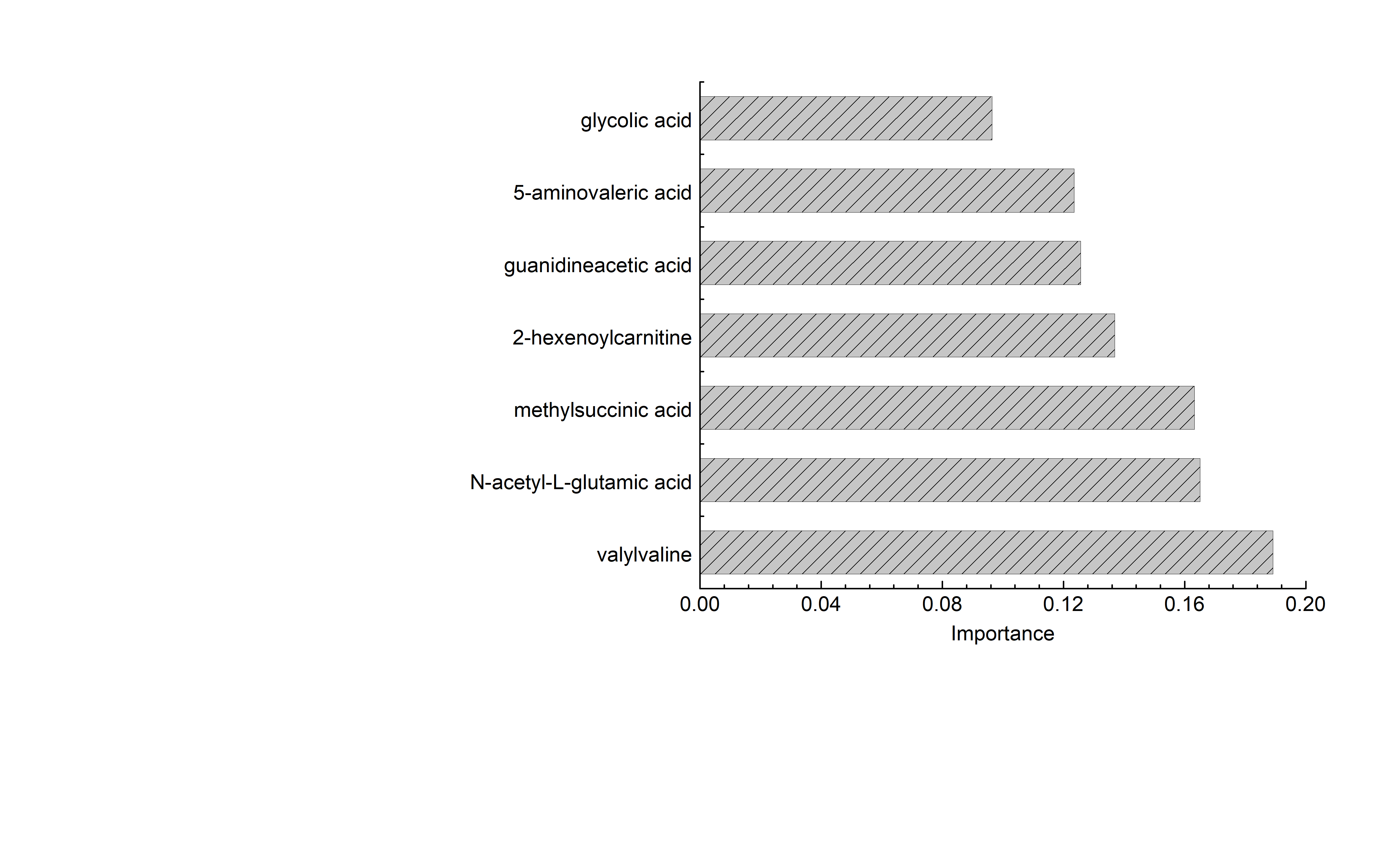

Supplement: Supplementary file 1 [file metabolites-13-00715-s001.zip › Supplementary File/Figure S3.png]

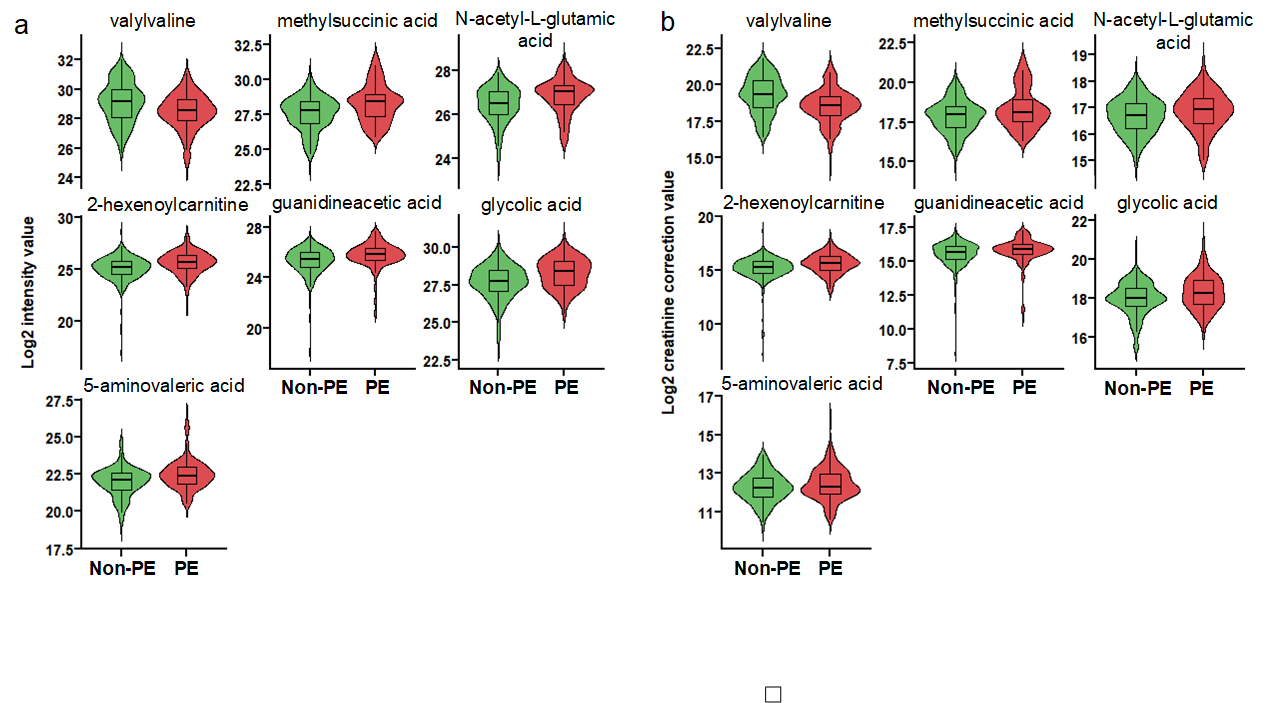

Supplement: Supplementary file 1 [file metabolites-13-00715-s001.zip › Supplementary File/Figure S4.png]

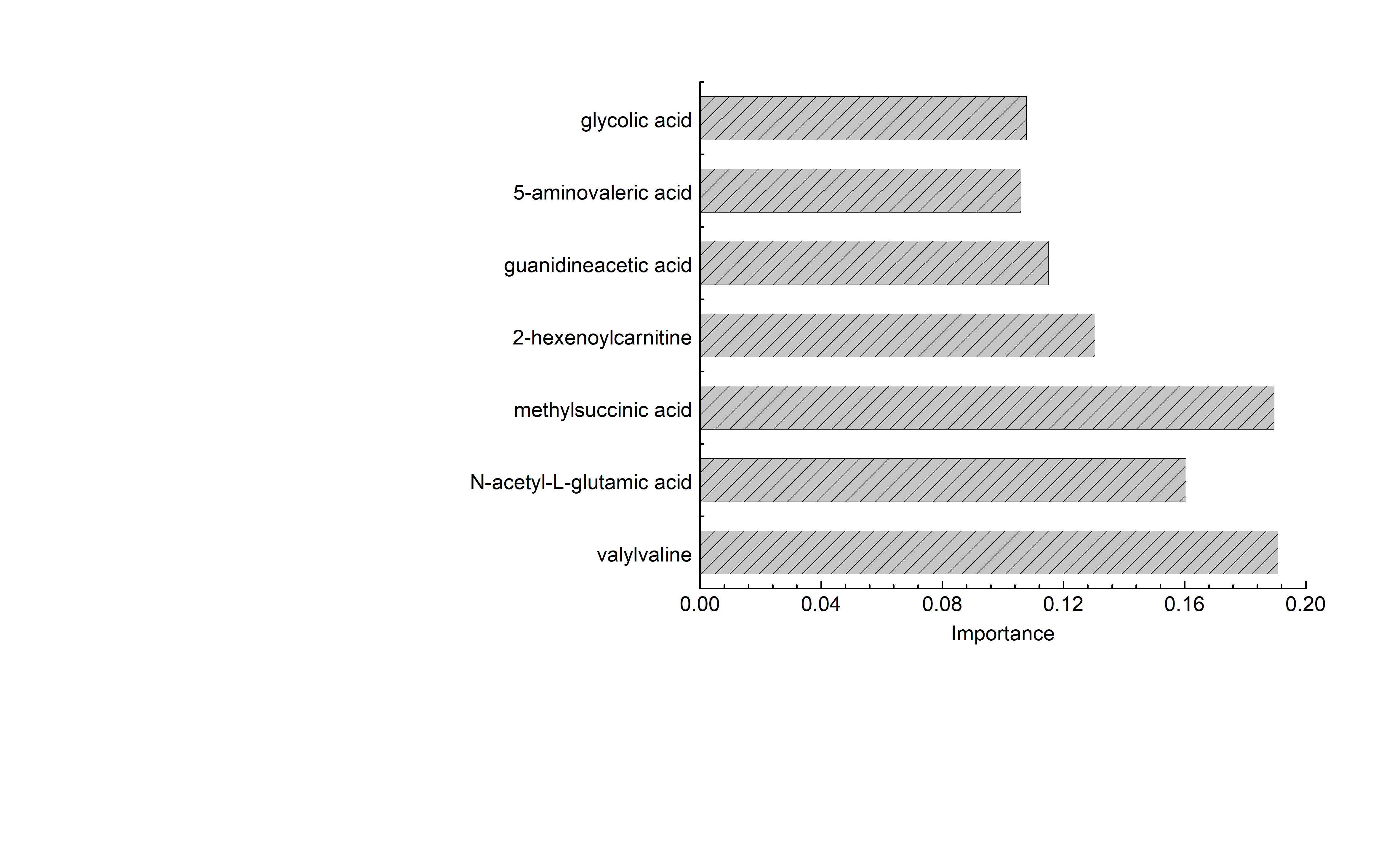

Supplement: Supplementary file 1 [file metabolites-13-00715-s001.zip › Supplementary File/Figure S5.png]

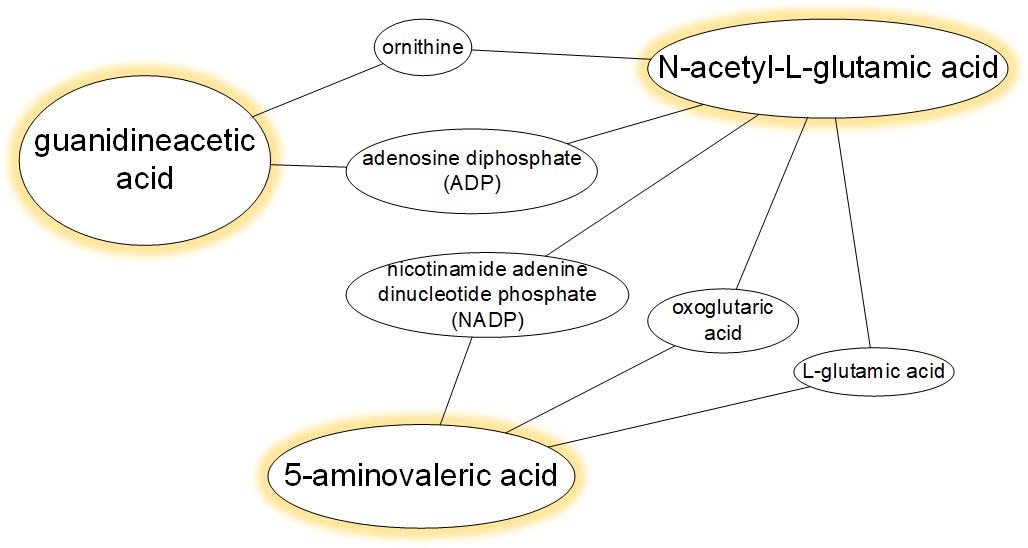

Supplement: Supplementary file 1 [file metabolites-13-00715-s001.zip › Supplementary File/Figure S6.png]
